# Mechanism of Action of Mindfulness-Based Interventions for Pain Relief—A Systematic Review

**DOI:** 10.1089/jicm.2023.0328

**Published:** 2024-12-06

**Authors:** Markus Ploesser, David Martin

**Affiliations:** Fakultät für Gesundheit (Department für Humanmedizin), Lehrstuhl für Medizintheorie, Integrative und Anthroposophische Medizin, Herdecke, Germany.

**Keywords:** systematic review, mindfulness, pain, mechanism of action

## Abstract

**Background:**

Currently, no systematic evidence synthesis of the mechanism of action of mindfulness-based approaches exists for pain conditions.

**Aim:**

To identify and synthesize experimental and clinical studies examining aspects of the mechanism of action of mindfulness for pain relief.

**Methods:**

The following databases and search interfaces were searched: Embase (via Embase.com) and Medline (via PubMed). Additional references were identified via bibliographies of included studies. The following were the inclusion criteria applied: (1) original studies published in peer-reviewed journals, (2) in adult populations that (3) examined the mechanism of action of mindfulness meditation on pain outcomes or (4) provided conclusions regarding the potential mechanism of action of mindfulness meditation. The studies were selected by two independent reviewers. Discrepancies were resolved by discussion.

**Results:**

A total of 21 studies published in English met the inclusion criteria, of which 5 studies were clinical studies, which included patients with chronic pain, and 16 studies used experimental pain induction. The investigation into brain mechanisms through functional magnetic resonance imaging and diffusion tensor imaging revealed mindfulness meditation’s ability to modulate brain activity, particularly in the anterior cingulate cortex, anterior insula, and orbitofrontal cortex, and to enhance structural and functional connectivity in regions associated with pain perception. Regarding the role of opioids, findings across five studies indicated that the analgesic effects of mindfulness are maintained even when opioid receptors are blocked, suggesting a nonopioidergic pathway for pain modulation. Pain perception studies highlighted that mindfulness practices foster pain acceptance and modify pain control beliefs, serving as key mediators in improving pain outcomes. For experienced versus novice mindfulness practitioners, results demonstrated that long-term practice enhances pain threshold and reduces pain unpleasantness through increased activity in salience and attentional control regions.

**Conclusion:**

This systematic review highlights mindfulness meditation as a multifaceted approach to pain management, utilizing mechanisms such as cognitive and emotional reappraisal, nonopioidergic pathways, and enhanced attention in control regions. It emphasizes the role of mindfulness in fostering pain acceptance and altering pain control perceptions, showcasing its broad impact on the neurological and experiential dimensions of pain. However, the predominance of studies on healthy subjects and methodological variations across experiments necessitates careful interpretation of the findings. The review calls for further research to explore the mechanisms of mindfulness in chronic pain populations more deeply, distinguishing the specific effects of mindfulness from nonspecific effects and expanding its applicability in clinical settings for chronic pain management.

## Introduction

Mindfulness-based therapies are defined as self-regulated attention strategies based on nonjudgmental awareness of the present moment.^1^ Mindfulness can be cultivated through a variety of meditation programmes and even untrained meditation activities. Within each of these strategies, there are numerous variations. Mindfulness-based stress reduction (MBSR) has been linked to a number of positive health outcomes.^2^ Different styles of meditation, such as sitting and moving meditation, are paired with behavioral therapy, daily assignment work, and a silent 1-day retreat in the MBSR program.^3^ Other mindfulness-based interventions grew out of MBSR, including mindfulness-based cognitive therapy (MBCT) for treating depression,^4,5^ mindfulness-oriented recovery enhancement for combating opioid abuse and chronic pain,^6^ and mindfulness-based relapse prevention, which has been shown to be effective for addiction treatment.^7^

Because mindfulness increases cognitive control^8^ and emotion regulation,^9^ it may have the ability to address several avenues in this complex network of brain systems which cause pain.^10^ Across clinical and experimental contexts, evidence shows that mindfulness meditation may be a promising alternative to reduce pain. The health-promoting effects of meditation are most prominent for pain and pain-related comorbidities, such as fibromyalgia^11^ and chronic low back pain,^12^ stress, depression, and anxiety.^13–15^

Previous narrative reviews propose that mindfulness alters the meaning, interpretation, and appraisal of nociceptive information, which are important considerations for stabilizing and long-term improvements in chronic pain symptomology, and which can potentially serve as a mechanism against the development of pain over time.^10,16^

Currently, no systematic evidence synthesis of the mechanism of action of mindfulness-based approaches exists for pain conditions. Therefore, the objective of this systematic review is to identify and synthesize experimental and clinical studies examining aspects of the mechanism of action of mindfulness for pain relief.

## Methods

English language articles were searched from database start up to January 2022 in MEDLINE (via Pubmed.com) and Embase (via Embase.com). To minimize the risk of selection bias, English abstracts of articles in other languages were screened to determine suitability for translation and inclusion into the review. Reference lists of reviews and obtained articles were screened for further studies to include. To obtain additional study information, authors of publications were contacted when necessary. So-called “gray literature” (i.e., Doctoral and Master dissertations) was searched through google scholar. Search terms included “mindfulness AND pain.”

The following were the inclusion criteria used during the screening process: (1) original studies published in peer-reviewed journals, (2) in adult populations that (3) examined the mechanism of action of mindfulness meditation on pain outcomes or (4) provided conclusions regarding the potential mechanism of action of mindfulness meditation. The criteria for exclusion were as follows: (1) articles that did not contain original research (i.e., reviews and meta-analyses, guidelines and/or protocols), (2) clinical trials focusing on treatment gains or efficacy for pain reduction, and (3) studies in languages other than English. The studies were selected by two independent reviewers. Data extraction was also performed by two independent reviewers. Discrepancies were resolved by discussion. All relevant data were synthesized in the table.

The protocol for this systematic review has not been published, and the review is not registered with a systematic review database.

## Results

**FIG. 1.**
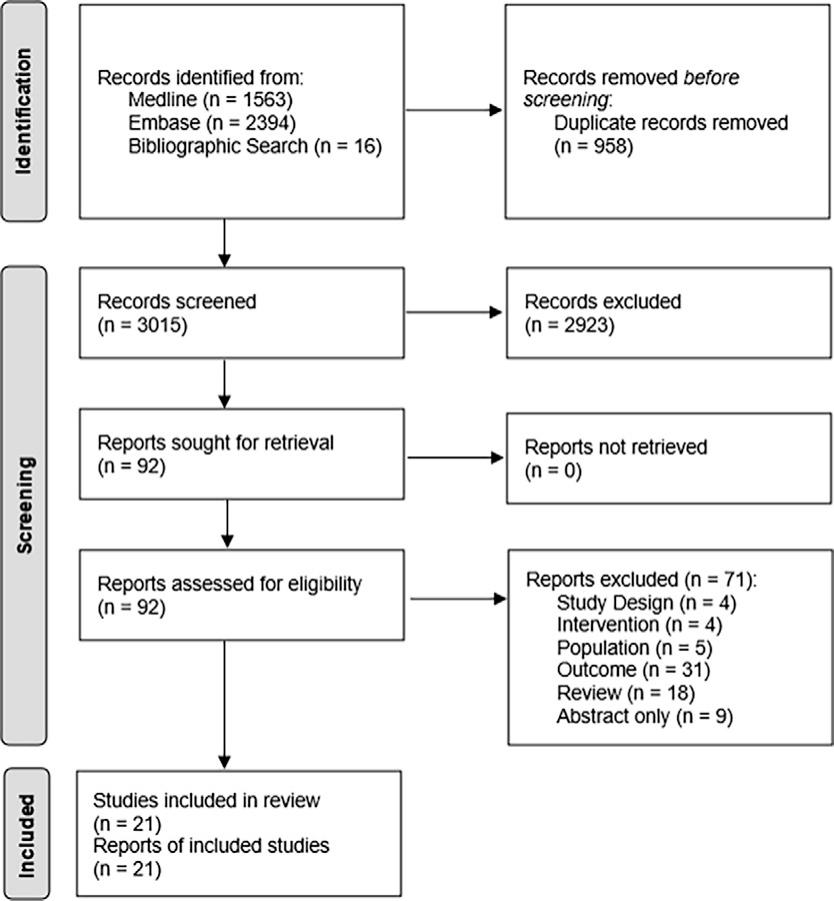
PRISMA flow diagram.

Figure 1 shows a flowchart for the selection of eligible studies. The search strategy initially identified 3957 studies through database searching and 16 additional studies through bibliographic searching. After removing duplicates (*n* = 958), 3015 articles were screened according to the inclusion criteria. If these were met, the full-text article was retrieved and screened in full for the analysis. A total of 21 studies published in English met the inclusion criteria and were selected for review.

The studies were classified into three broad categories, according to outcomes as follows: (1) neurological outcomes, (2) outcomes comparing experienced versus novice mindfulness practice, and (3) pain perception outcomes, including studies on the role of opioids and other. A summary of the main characteristics of each study, including participants, study designs, sample size, control conditions, outcomes, and conclusions can be obtained from Table 1.

**Table 1.** Study Characteristics and Outcomes

Study	Study design	Study sample	Main outcomes	Experimental source of pain	Intervention	Control	Main findings	Conclusions
Neurological outcomes								
Kober et al., 2019^17^	Experimental, randomized	Healthy, meditation-naïve adults, *N* = 17	Negative affect, pain outcomes, functional MRI	Induced, thermal stimuli (up to 49°C)	Thermal stimulation plus 30 neutral and 30 negative images plus ACCEPT condition (mindfully accept the experience as it is)	Thermal stimulation plus 30 neutral and 30 negative images plus REACT condition (react naturally, whatever the response might be)	Mindful acceptance led to significant reductions in negative affect and reported pain, with a main effect of instruction showing lower negative affect for both negative images (*p* < 0.001, *d* = 1.14) and painfully hot temperatures (*p* < 0.001, *d* = 1.51) when participants used ACCEPT versus REACT instructions (*p* < 0.001). Functional MRI results indicated that ACCEPT instructions notably decreased activity in the right amygdala for negative images (*p* < 0.001) and in pain-related brain regions including the dACC, medial frontal gyrus, anterior and posterior insula, thalamus, and cerebellum during painful heat exposure. The ACCEPT-hot condition resulted in a significantly lower NPS response compared with REACT-hot (*p* = 0.02, *d* = 0.65), demonstrating a 26% reduction in the NPS, highlighting the effectiveness of mindful acceptance in modulating the brain’s pain response.	Momentary mindful acceptance regulates emotional intensity by changing initial appraisals of the affective significance of stimuli
Mioduszewski et al., 2020^18^	Clinical, randomized	Breast cancer survivors with chronic neuropathic pain, *N* = 23	Pain outcomes, white matter integrity by diffusion tensor imaging		MBSR	Waiting list	The MBSR group showed significant increases in FA in left subcortical regions (external capsule, uncinate fasciculus, amygdala, hippocampus cingulum) and the left sagittal stratum, indicating improved white matter integrity Pain severity and interference scores significantly decreased within the MBSR group from pre- to postscan (pain severity: prescan mean = 4.451, postscan mean = 3.736, *p* = 0.015; pain interference: prescan mean = 4.359, postscan mean = 3.351, *p* = 0.035). Negative correlations were found between FA values in the left sagittal stratum and both pain severity (*r* = −0.49, *p* < 0.05) and pain interference scores (*r* = −0.436, *p* < 0.05) postscan, linking higher FA to lower pain scores.	MBSR training may enhance the integrity of cerebral white matter that coincides with a reduction in pain perception
Seminowicz et al., 2020^19^	Clinical, randomized	Adults with episodic migraine, *N* = 98	Reduction in headache days, pain ratings, functional MRI		MBSR	SMH	At week 20, the MBSR group reported significantly fewer headache days (mean = 4.6, 95% CI [3.6–5.6]) compared with the SMH group (mean = 6.0, 95% CI [4.9–7.0], *p* = 0.04), with 52% of MBSR participants classified as treatment responders (≥50% reduction in headache days) versus 23% in the SMH group (*p* = 0.004), yielding an NNT of 3.4. MBSR group also reported reduced HIT-6 scores (2.0 [95% CI 1.1–2.9]) compared with the SMH group (3.7 [95% CI 2.7–4.7]; *p* = 0.04) at week 20, indicating a significant improvement in headache-related disability. Functional MRI analyses revealed significant treatment-by-time interactions, with the MBSR group showing decreased activation in the bilateral cuneus and right parietal operculum during a cognitive challenge at week 20 compared with the SMH group, indicating changes in cognitive networks associated with MBSR.	In episodic migraine, MBSR showed superior treatment effects compared with an active control, with significant reductions in headache frequency. Brain changes in the MBSR group were seen in the pattern of functional connectivity and activation during a challenging cognitive task that are consistent with increased cognitive efficiency.
Smith et al., 2021^20^	Clinical, randomized	Breast cancer survivors with chronic neuropathic pain, *N* = 23	Functional MRI, pain outcomes		MBSR	Waiting list	Post-MBSR intervention, significant increases in FC between the PCC and ACC/medial PFC were observed in the MBSR group compared with controls, reflecting enhanced connectivity within the DMN associated with reduced pain severity (PCC–ACC/medial PFC FC increase: *r* = −0.57, *p* < 0.005). Within the MBSR group, pain severity and pain interference scores significantly decreased after the 8-week training (pain severity: prescan mean = 4.45, postscan mean = 3.74, *p* = 0.02; pain interference: prescan mean = 4.356, postscan mean = 3.35, *p* = 0.04), indicating an effective reduction in perceived pain levels. Additionally, changes in FC between the PCC and bilateral precentral gyrus positively correlated with changes in pain severity scores (*r* = 0.51, *p* = 0.014), suggesting that increases in this specific FC were associated with higher pain severity scores post-MBSR.	Empirical evidence of a change in brain activity following MBSR intervention associated with changes in the subjective experience of pain
Su et al., 2016^21^	Clinical, pre–post-test	Adult with moderate to severe pain (*n* = 18) and no to mild pain (*n* = 16); total *N* = 34	Functional MRI, pain outcomes	N/A	MBSR	N/A	After a 6-week mindfulness training, the pain-afflicted group experienced significant reductions in pain perception, as shown by a main effect of time (*F* = 8.91, *p* < 0.01) and an interaction of time × group (*F* = 5.28, *p* < 0.05) in the SFMPQ scores, with posthoc comparisons indicating a significant change in the composite score of the SFMPQ in the pain-afflicted group (*t* = 3.05, *p* < 0.01). FC analysis between the AIC and the daMCC revealed a significant interaction effect, with increased connectivity post-training in the pain-afflicted group (*t* = 2.68, *p* < 0.01) and a significant negative correlation between AIC–daMCC connectivity and SFMPQ scores (*r* = −0.48, *p* < 0.05), suggesting that increased AIC–daMCC connectivity was associated with lower pain scores. There were no significant changes in pain scores or AIC–daMCC connectivity in the control group (*t* = 1.08, *p* = 0.30), and no significant correlations between AIC–daMCC connectivity and the DPQ (*r* = −0.05, *p* = 0.79) or KIMS scores (*r* = −0.07, *p* = 0.68) were observed.	Mindfulness training can modulate the brain network dynamics underlying the subjective experience of pain.
Zeidan et al., 2011^22^	Experimental, randomized	Healthy volunteers, *N* = 18	Functional MRI, pain outcomes	Induced, noxious (49°C) or neutral (35°C) thermal stimulus	Noxious stimulus (49°C), mindfulness meditation	Neutral stimulus (35°C), mindfulness meditation	Meditation significantly reduced pain intensity by 40% and pain unpleasantness by 57% after 4 days of meditation training, with substantial decreases in VAS pain intensity (*p* < 0.001) and pain unpleasantness ratings (*p* < 0.001) observed during the second MRI session compared with rest. Meditation-related brain activity showed bilateral activation in regions corresponding to the nose and throat in the primary somatosensory cortex (SI), posterior insula, and secondary somatosensory cortex (SII), as well as increased activity in areas associated with interoceptive attention like the AI and ACC. Additionally, individuals with the greatest reductions in pain intensity exhibited the largest meditation-induced activation of the right AI and bilateral ACC, whereas the greatest reductions in pain unpleasantness were associated with the highest activation of the OFC and the most significant deactivation of the thalamus.	Meditation engages multiple brain mechanisms that alter the construction of the subjectively available pain experience from afferent information
Zeidan et al., 2015^23^	Experimental, randomized	Healthy volunteers, *N* = 80	Functional MRI, pain outcomes	Induced, thermal stimuli (up to 49°C)	Thermal stimuli, placebo cream (was described to participants as to be tested for its pain-reducing properties); mindfulness meditation, sham mindfulness meditation	Thermal stimuli, listening to an unrelated neutral audio recording	Mindfulness meditation, placebo, and sham mindfulness meditation all significantly reduced pain intensity ratings from pre- to postmanipulation, with mindfulness meditation showing the greatest reduction in pain intensity (27% reduction, *p* = 0.002) and unpleasantness (44% reduction, *p* < 0.001) compared with placebo (pain intensity reduction 11%, *p* = 0.028; pain unpleasantness trend toward reduction 13%, *p* = 0.054) and sham mindfulness (pain intensity reduction 8%, *p* = 0.044; pain unpleasantness reduction 27%, *p* = 0.003). The significant group × manipulation interaction on pain ratings was most notable for mindfulness meditation, significantly outperforming placebo (*p* = 0.032), sham mindfulness (*p* = 0.030), and control conditions (pain intensity: *p* < 0.001; pain unpleasantness: *p* < 0.001) in reducing both pain intensity and unpleasantness ratings. Neuroimaging findings revealed that mindfulness-meditation-related pain relief was associated with greater activation in brain regions associated with the cognitive modulation of pain, including the OFC, sgACC, and AI, whereas placebo analgesia involved activation of the dorsolateral PFC and deactivation of sensory processing regions. Sham mindfulness meditation-induced analgesia was not correlated with significant neural activity but was associated with reductions in respiration rate.	Mindfulness-related pain relief is mechanistically distinct from placebo analgesia, suggesting the existence of multiple, cognitively driven, supraspinal mechanisms for pain modulation
*Experienced versus novice mindfulness practice*								
Lutz et al., 2013^24^	Experimental, pre–post-test study	Healthy adults, *N* = 28 (14 long-term mindfulness practitioners + 14 without mindfulness experience)	Pain unpleasantness, pain intensity, functional MRI	Induced, thermal stimuli to left wrist (up to 49°C)	Thermal stimuli to left wrist (up to 49°C); meditation practice (focused attention, open monitoring)	N/A	Expert meditators reported significantly less pain unpleasantness compared with novices without differences in pain intensity ratings, driven by lower unpleasantness ratings (*p* = 0.001) among experts. Neuroimaging results revealed enhanced activity in the salience network, particularly in the dorsal AI and the aMCC, during pain processing among expert meditators compared with novices. This increased activity was associated with the experts’ reduced pain unpleasantness ratings but not with changes in pain intensity. Meditation expertise also modulated baseline brain activity before pain, with experts showing reduced activity in anxiety-related regions, including the left AI and aMCC, suggesting less anticipatory anxiety. The reduced baseline activation in left AI was negatively correlated with lifetime meditation experience (*r* = −0.63, *p* < 0.05), highlighting the cumulative effect of meditation practice on brain function related to pain and anxiety anticipation.	Cultivating experiential awareness downregulates anticipatory representation of aversive events and increases the recruitment of attentional resources during pain,
Perlman et al., 2010^25^	Experimental, pre–post-test study	Healthy adults, *N* = 19 (9 long-term mindfulness practitioners + 10 without mindfulness experience)	Pain unpleasantness, pain intensity	Induced, thermal stimuli to left wrist (up to 49°C)	Thermal stimuli to left wrist (up to 49°C); meditation practice (focused attention, open monitoring)	N/A	Long-time meditators, compared with novices, had a significant reduction of self-reported unpleasantness (*p* = 0.013), but not intensity, of painful stimuli while practicing open monitoring. No significant effects were found for focused attention.	Training of specific cognitive strategies can affect the subjective unpleasantness of a sensory experience separately from the intensity
*Pain outcomes*								
*Role of opioids*								
Case et al., 2021^26^	Experimental, randomized	Pain-free participants, *N* = 78	Expectations about mindfulness and pain ratings	Induced, thermal stimuli (up to 49°C), plus either naloxone (opioid antagonist) or saline	Mindfulness meditation, thermal stimuli plus naloxone (opioid antagonist)	Rest, thermal stimuli plus saline	Before the intervention, expectations for pain relief were significantly higher in the mindfulness group compared with the book-listening group (*p* < 0.001). Postintervention, mindfulness meditation significantly predicted reductions in pain intensity (*p* = 0.002) and unpleasantness (*p* = 0.040) in the naloxone group. This effect was notably stronger than in the saline group, where no significant correlation between expectations and pain relief was observed (intensity: *p* = 0.91; unpleasantness: *p* = 0.36). After the intervention, mindfulness meditation led to significant reductions in pain intensity and unpleasantness, which were not reliant on endogenous opioids. This was evident from the lack of significant differences in pain reductions between the mindfulness + naloxone (intensity: −24%; unpleasantness: −33%) and mindfulness + saline (intensity: −21%; unpleasantness: −36%) groups.	There is a significant role for expectations in mindfulness-based pain-relief and a nonopioidergic pain modulatory pathway to underlie the pain-relieving effects of mindfulness meditation
Esch et al., 2017^27^	Experimental, randomized	Healthy adults, *N* = 31	Pain tolerance, attention performance	Induced, tourniquet test, plus either naloxone (opioid antagonist) or saline	Mindfulness meditation, tourniquet test plus either naloxone (opioid antagonist) or saline	No intervention, tourniquet test plus either naloxone (opioid antagonist) or saline	Initially, the meditation group increased their pain tolerance by 2 min 1 s postintervention, whereas the control group gained 5 min 37 s, with no significant difference between groups (*p* = 0.14). Naloxone administration did not significantly change pain tolerance compared with placebo in both groups, with participants tolerating pain slightly longer under placebo than naloxone, but the difference was not statistically significant (*p* = 0.16). Participants improved in reaction time postintervention (from 496 ms to 471 ms, *p* < 0.001), but there were no significant group differences in attention indicators. Error scores slightly decreased after the intervention, indicating improved attention performance, with meditators showing a trend toward fewer errors. Increases in self-attributed mindfulness did not significantly correlate with improved pain tolerance within the meditation group alone. However, when considering the whole sample, there was a significant medium-sized correlation between the increase in self-attributed mindfulness and pain tolerance improvement (*p* = 0.046), indicating a positive relationship between mindfulness and the ability to tolerate pain.	Higher pain tolerance through meditation could not be confirmed. A possible impact of opioidergic signaling in meditation and pain physiology could not be established.
May et al., 2018^28^	Experimental, randomized (crossover)	Healthy, experienced meditation practitioners, *N* = 32	Pain ratings	Induced, electrical stimulation, plus either naloxone (opioid antagonist) or saline	Electrical stimulation, plus naloxone (opioid antagonist), open monitoring meditation	Electrical stimulation, plus saline, open monitoring meditation	Pain ratings during meditation were significantly lower than at baseline for both intensity (*p* < 0.001) and unpleasantness (*p* < 0.001). Naloxone had a significant effect on pain intensity (*p* = 0.050) but not on pain unpleasantness. Pain intensity decreased from 6.86 to 6.41 under saline and from 6.73 to 5.53 under naloxone, with effect sizes of 0.46 and 1.08, respectively. Pain unpleasantness decreased from 4.96 to 3.98 under saline and from 4.87 to 2.95 under naloxone, with effect sizes of 0.68 and 1.38, respectively. Comparisons between saline and naloxone during meditation revealed significantly lower pain intensity (*p* = 0.004) and pain unpleasantness (*p* = 0.002) under naloxone, indicating that naloxone not only failed to eliminate meditation analgesia but also enhanced it.	Long-term meditation practice does not rely on endogenous opioids to reduce pain. Naloxone’s blockade of opioid receptors enhanced meditation analgesia; pain ratings during meditation were significantly lower under naloxone than under saline
Sharon et al., 2016^29^	Experimental, randomized (crossover)	Healthy experienced mindfulness meditation practitioners, *N* = 15	Pain and unpleasantness	Induced, cold stimulus, plus either naloxone (opioid antagonist) or saline	Cold stimulus, plus naloxone (opioid antagonist), mindfulness meditation	Cold stimulus, plus saline, mindfulness meditation	Significant reductions in pain (from 6.11 ± 0.46 to 4.21 ± 0.5, *p* < 0.001) and unpleasantness scores (from 5.8 ± 0.47 to 3.4 ± 0.44, *p* < 0.001) after natural mindfulness meditation compared with baseline Similar significant reductions were observed after placebo administration for pain (from 6.14 ± 0.48 to 5 ± 0.66, *p* < 0.01) and unpleasantness scores (from 5.71 ± 0.48 to 4.28 ± 0.58, *p* < 0.01). No significant reductions were found after naloxone administration for pain (from 6 ± 0.47 to 5.2 ± 0.56, *p* = 0.1) or unpleasantness scores (from 5.9 ± 0.5 to 4.78 ± 0.5, *p* = 0.07).	Meditation involves endogenous opioid pathways, mediating its analgesic effect
Wells et al., 2020^30^	Experimental, randomized	Healthy, pain-free, and meditation-naïve participants, *N* = 60	Pain ratings	Thermal stimulus (heat)	Thermal stimulus plus either: mindfulness meditation, sham mindfulness meditation, or slow breathing exercise, plus naloxone (opioid antagonist) in each group (crossover)	Thermal stimulus plus either: mindfulness meditation, sham mindfulness meditation, or slow breathing exercise, plus saline in each group (crossover)	Mindfulness meditation and slow-paced breathing demonstrated opioid-independent analgesia, with no significant changes in pain intensity (*p* = 0.79; CI −12% to 16%) and pain unpleasantness (*p* = 0.76; CI −16% to 21%) ratings between saline and naloxone sessions. Specifically, mindfulness meditation altered pain intensity from −7% to −5% and pain unpleasantness from −18% to −15%, whereas slow-paced breathing affected pain intensity from −10% to −11% and pain unpleasantness from −11% to −18%. Conversely, sham mindfulness meditation’s effect on pain unpleasantness was significantly reversed by naloxone (*p* = 0.02; CI −3% to 38%), changing from −8% (saline) to +12% (naloxone).	Attention to breath reduces pain independent of endogenous opioids
Zeidan et al., 2016^31^	Experimental, randomized	Healthy, pain-free, and meditation-naïve volunteers, *N* = 95	Pain ratings	Induced, thermal stimuli (up to 49°C), plus either naloxone (opioid antagonist) or saline	Mindfulness meditation, thermal stimulus, plus naloxone (opioid antagonist)	Listening to an unrelated neutral audio recording, thermal stimulus, plus saline	Mindfulness meditation, compared with control, significantly reduced pain intensity by 21% (*p* < 0.001) and pain unpleasantness by 36% (*p* < 0.001) with saline, showing meditation’s analgesic effect. Naloxone infusion did not reverse these effects; there were no significant differences in pain intensity (*p* = 0.69) or unpleasantness reductions (*p* = 0.75) between meditation with naloxone and saline. Meditation under naloxone notably decreased pain intensity by 24% and unpleasantness by 33% compared with controls.	Mindfulness meditation does not rely on endogenous opioidergic mechanisms to reduce pain
*Pain perception*								
Adler-Neal et al., 2020^32^	Experimental, randomized	Healthy volunteers, *N* = 62	Pain outcomes, change in HRV	Induced, thermal stimuli to right calf (up to 49°C)	Mindfulness meditation, thermal stimuli	Sham mindfulness (breathing exercises), thermal stimuli	Mindfulness-induced reductions in pain unpleasantness were associated with higher HRV compared with sham mindfulness, with a significant group × HRV interaction (*p* = 0.04), and trends toward significance within the mindfulness group (*p* = 0.07) but not within the sham mindfulness group (*p* = 0.11).	Mindfulness-based meditation engages distinct mechanisms from sham mindfulness meditation to reduce pain
Day and Thorn., 2016^33^	Clinical, randomized	Patients with headache, *N* = 24	Pain and mediators	Headache	MBCT	Wait list control	Pain acceptance emerged as a significant mediator of the group-interference relation (*p* < 0.05). Mediation models examining acceptance subscales showed nuances in this effect, with activity engagement emerging as a significant mediator (*p* < 0.05).	Pain acceptance, and specifically engagement in valued activities despite pain, may be a key mechanism underlying improvement in pain outcome during an MBCT for headache pain intervention
Day et al., 2020^34^	Clinical, randomized	Adults with chronic low back pain, *N* = 69	Pain interference, pain intensity	N/A	Mindfulness meditation	Cognitive therapy, mindfulness-based cognitive therapy	Pain control beliefs and catastrophizing significantly correlated with reduced pain interference across the three conditions, with small to medium effect sizes; therapeutic alliance significantly correlated with pain intensity improvement (therapeutic alliance effect on pain intensity *p* = 0.019).	Change in perceived pain control and pain catastrophizing emerged as potential “metamechanisms” that might be a shared pathway that contributes to improved pain-related outcomes across treatments
Reiner et al., 2016^35^	Experimental, randomized	Undergraduate students, *N* = 40	Response pattern over time to tonic heat pain	Induced, thermal stimuli (up to 49°C)	Mindfulness meditation, thermal stimuli	No intervention, thermal stimuli	The mindfulness meditation group showed increased heat pain threshold (*p* < 0.001) and more rapid attenuation of pain intensity for tonic pain stimuli.	A brief mindfulness meditation intervention appears to affect perception of experimental pain both by increasing pain threshold and accelerating modulation of response
Smith et al., 2017^36^	Experimental, randomized	University undergraduates, *N* = 63	Pain ratings	Induced, thermal stimulus (cold)	Thermal stimulus plus either deep breathing, progressive muscle relaxation	Thermal stimulus plus control condition (no explicit instruction to watch breathing)	No differences were observed in participants’ pain tolerances or self-reported pain ratings during the cold pain task or in their physiological responses, including HR, HRV, and respiration (*p* = 0.112 for HR; *p* = 0.102 for HRV; *p* = 0.461 for respiration). Individual differences in physiological functioning were not related to pain tolerance or pain ratings, with no significant condition effects (hazard ratio = 0.991, *p* = 0.962) or changes in physiological measures influencing pain tolerance.	The mechanisms through which mindfulness exerts its effects on pain are more complex than through physiological changes brought about by altering breathing or muscle tension.
Wang et al., 2019^37^	Experimental, randomized	Healthy college students without prior mindfulness experience; *N* = 119	Pain intensity, tolerance, distress, threshold, and endurance time	Induced, place hand into ice-cold water (cold pressure test)	Component of mindfulness meditation (pain acceptance, pain attention, combination of both), thermal stimulus	Neutral reading material in general silence, thermal stimulus	The combined acceptance and attention group increased pain endurance and tolerance after mindfulness meditation, with significant main effects of time for endurance (*p* < 0.001) and tolerance (*p* = 0.001). The acceptance group had significantly longer pain endurance (*p* < 0.001, Cohen’s *d* = 0.635) and tolerance times (*p* < 0.001, Cohen’s *d* = 0.634) than the attention and control groups in the post-test.	Acceptance of pain is more important than attention to pain in the early stages of pain management

ACC, anterior cingulate cortex; AI, anterior insula; AIC, anterior insular cortex; aMCC, anterior midcingulate cortex; CI, confidence interval; daMCC, dorsal anterior midcingulate cortex; DMN, default mode network; DPQ, Dallas Pain Questionnaire; FC, functional connectivity; HF, high frequency; HIT-6, headache impact test; HR, heart rate; HRV, heart rate variability; KIMS, Kentucky Inventory of Mindfulness Skills; MBSR, mindfulness-based stress reduction; MBCT, mindfulness-based cognitive therapy; MRI, magnetic resonance imaging; N/A, not applicable; NNT, number needed to treat; NPS, neural pain signature; OFC, orbitofrontal cortex; PCC, posterior cingulate cortex; PFC, prefrontal cortex; SFMPQ, Short Form McGill Pain Questionnaire; sgACC, subgenual anterior cingulate cortex; SMH, stress management for headache.

Based on the selected articles, seven studies reported on neurological outcomes,^17–23^ two studies examined the difference in pain outcomes with experienced versus novice mindfulness practitioners,^24,25^ six studies investigated pain outcomes and the role of opioids,^26–31^ and six studies reported on outcomes related to general perceptions of pain (Table 1).^32–37^

Out of the 21 studies included, 5 studies were clinical studies which included patients with chronic pain, such as low back pain,^34^ headache,^19,33^ and chronic neuropathic pain.^18,20^ Of the 16 studies which used pain induction, 13 were randomized studies^17,22,23,26–32,35–37^ and 3 were pretest-posttest studies.^21,24,25^ Sample sizes ranged from *n* = 119^36^ to *n* = 15,^29^ of which 15 studies had a study population of <100 (Table 1).

### Studies on neurological outcomes of pain management with mindfulness

A total of seven studies examined the mechanism of action of mindfulness based on neurological outcomes.^17–23^

Out of these, six studies used functional magnetic resonance imaging (MRI) and one study conducted the test by diffusion tensor imaging.^18^ Results indicate that meditation appears to engage multiple brain mechanisms that alter the construction of the subjectively available pain experience (Table 1).

Clinical studies, like those conducted by Mioduszewski et al. (2020), Seminowicz et al. (2019), and Smith et al. (2020),^18–20^ have demonstrated through diffusion tensor imaging and functional MRI that mindfulness meditation enhances structural and functional connectivity (FC) in the brain, particularly increasing fractional anisotropy in the uncinate fasciculus, amygdala, hippocampus, and improving connectivity between the posterior cingulate and medial prefrontal regions. These changes correlate with reductions in pain perception and severity in conditions like episodic migraine and chronic neuropathic pain (Table 1).

In contrast, experimental studies by Kober et al. (2019), Su et al. (2016), Zeidan et al. (2011), and Zeidan et al. (2015),^17,21–23^ utilizing functional MRI in healthy subjects, have identified mindfulness meditation’s activation of the anterior cingulate cortex (ACC), anterior insula (AI), and orbitofrontal cortex (OFC), alongside reduced activation in the primary somatosensory cortex and amygdala, outlining a detailed map of neural mechanisms that mediate the pain-relieving effects of mindfulness (Table 1). For example, the experimental studies by Zeidan et al. (2011, 2015) demonstrated that mindfulness meditation significantly reduced both pain intensity and unpleasantness ratings, linking these effects to changes in brain activity (Table 1).^22,23^

### Studies examining experienced versus novice mindfulness practice

In comparing experienced mindfulness practitioners with meditation-naïve individuals, Lutz et al. (2013) and Perlman et al. (2010) found that although pain intensity remained consistent across groups, experienced meditators reported significantly less pain unpleasantness (Table 1).^24,25^

### Studies investigating the role of opioids in pain outcomes

Out of the six studies investigating the role of opioids in pain outcomes with mindfulness, five reported findings indicative of a nonopioidergic pathway facilitating the pain-relieving effects of mindfulness meditation.^26–28,30,31^ Specifically, these studies found that mindfulness meditation reduced pain intensity and unpleasantness in participants, even when opioid receptors were blocked by naloxone, highlighting a mechanism independent of the body’s endogenous opioid system. For instance, May et al. (2018) observed enhanced meditation analgesia under naloxone blockade, suggesting that long-term meditation practice strengthens nonopioidergic pain modulation.^28^ Similarly, Zeidan et al. (2016) reported that mindfulness meditation activated brain regions associated with cognitive modulation of pain, such as the OFC and ACC, without the involvement of opioidergic pathways (Table 1).^31^

### Studies on general pain perception

Mindfulness meditation was found to enhance pain management through mechanisms such as increased pain acceptance, changes in pain control beliefs, and reductions in pain catastrophizing across both clinical^33,34^ and experimental studies,^32,35–37^ with one experimental study (Adler-Neal et al., 2020) demonstrating that mindfulness also led to physiological changes, such as increased heart rate variability (HRV), associated with reduced pain unpleasantness (Table 1).^32^

## Discussion

According to this review of the existing literature, mindfulness meditation for pain relief appears to have favorable effects via multiple modes of action; however, the major mechanism via which the therapeutic effect is supplied could not be clearly identified from the current research.

The results of this review show that mindfulness meditation-based pain alleviation engages a variety of mechanisms that are representative of reappraisal processes that are not relying on a single brain mechanism to alleviate pain. These pathways elucidate how mindfulness meditation influences pain perception and management through a combination of neurological, cognitive, and experiential changes, distinct from opioid-based mechanisms. The included brain studies have shown that mindfulness meditation affects pain perception by regulating emotional intensity and altering brain activity, reducing negative affect and pain by modulating activity in the amygdala and other pain-related brain regions, leading to a significant decrease in the neural pain signature. This suggests that mindfulness regulates pain through cognitive and emotional reappraisal mechanisms. However, the majority of these studies presented clear hypotheses but generally lacked clear primary outcome parameters and did not conduct sample size calculations. This suggests a methodological weakness in these studies, potentially affecting the clarity of their objectives and the statistical power to detect meaningful effects.^17,18,20–23^

The results of the studies investigating the role of opioids in pain relief indicate that the pain-relieving effects of mindfulness meditation do not rely on endogenous opioids. The majority of studies using naloxone, an opioid antagonist, have shown that mindfulness meditation’s analgesic effects are maintained even when opioid receptors are blocked, pointing to a nonopioidergic pain modulatory pathway.^26–31^

The clinical studies examining general pain perception reveal that pain acceptance and changing pain perception and control beliefs are key mediators in MBCT for improving outcomes in patients with headache and chronic low back pain.^33,34^ Meanwhile, experimental investigations highlight distinct mechanisms of mindfulness meditation in pain modulation, including increased HRV, enhanced pain threshold, more complex cognitive and affective pathways beyond physiological changes, and the pivotal role of acceptance in pain management.^32,35–37^

Additionally, the accumulated experience of meditation practice significantly affects the perception and modulation of pain. Experienced meditators show reduced pain unpleasantness and enhanced activity in brain regions associated with salience and attentional control, indicating that long-term practice cultivates an experiential awareness that downregulates anticipatory anxiety and recruits attentional resources more efficiently during pain processing.^24,25^

Pain is a complex and subjective conscious experience that is shaped and modulated by a variety of sensory, cognitive, and affective factors, such as mood, psychological disposition, learning, expectations and prepain cognitive states (e.g., expectations; anxiety).^9,10,38–42^ Additionally, the setting in which pain arises continues to have a significant impact on the subjective experience of pain. Previous experiences, expectations, attitude, training, desires, sensitization/habituation, and other cognitive factors can all drastically increase or decrease pain.^10,42–47^ Peripheral primary afferents register nociceptive sensory events at the site of injury/tissue destruction, and subsequently convey this information to the dorsal horn of the spinal cord. Nociceptive information travels from the spinal cord to the brain contralateral to the site of pain, primarily via the spinothalamic pathway. Feedback connections between lower-level sensory regions, such as the parabrachial nucleus, periaqueductal gray matter (PAG), thalamus, and main and secondary somatosensory (SI) cortices, are used to process nociceptive input,^9,10,38–42^ with the ascending nociceptive information then being sent to the posterior and anterior insular cortices (AICs), where it aids with pain appraisal.^48^ The activation of higher-order brain areas such as the ACC, dorsal ACC (dACC), and prefrontal cortex (PFC) facilitates the contextual meaning of pain.^10,48,49^

Previous research has shown that mindfulness may be a promising intervention at reducing pain,^50–53^ and the results of the present systematic review concur with previous reviews that it does so via involving processes that reflect changes in one’s relationship with pain.^3,10,16^ The identified studies on the comparison of novice (short-term) versus experienced (long-term) mindfulness practice^24,25^ indicate that with more experience, the mechanisms enabling mindfulness-based pain treatment will grow more impactful, in contrast to standard pain medicines, which show rising tolerance and efficacy plateaus.^54–56^

These results are in agreement with other authors, implicating that the brain processes that underpin mindfulness-induced pain reduction alter depending on the level of meditation, where mindfulness-based pain relief is associated with higher order (OFC and rostral ACC) regulation of low-level nociceptive neural targets (thalamus and primary somatosensory cortex) after brief mindfulness-based mental training, suggesting an engagement of unique reappraisal mechanisms.^3,10,16^ Mindfulness-based pain alleviation, on the other hand, is associated with prefrontal deactivation and increased activation of somatosensory cortical regions after extensive training, suggesting an ability to minimize evaluations of emerging sensory stimuli.^3,10,16^

Zeidan et al. (2016)^10^ postulates that activation of the OFC may help the thalamic reticular nuclei generate inhibitory connections, reducing nociceptive information throughout the cortex (evidenced by reductions in thalamic, PAG, and SI activation). Neuroimaging data adds to the understanding of these events. Through alterations in executive attention, novice meditators use higher-order brain areas (OFC, subgenual ACC [sgACC], AI) to downregulate ascending nociceptive input at the thalamus level. As a result, following a short period of mental training, meditation has an effect on sensory and affective pain responses.^10^ Long-term meditation practice, on the contrary, is associated with much increased activation in sensorimotor areas and inactivation of appraisal-related brain regions. Long-term meditation practitioners’ decoupling between sensory experience and meaning and/or contextualization of what pain means to them provides evidence that meditation’s analgesic effects can be developed and enhanced with more practice, which is important for those seeking long-term narcotic-free pain relief.^24,25^

The results of this present systematic review show that meditation was observed to significantly lower pain intensity and unpleasantness ratings when compared with rest and control groups, regardless of naloxone or placebo-saline delivery. The endogenous opioidergic system includes cannabinoid, serotonergic, dopaminergic, cholecystokinin, adrenergic, and other neurochemical systems (i.e., vasopressin). Endogenous opioidergic systems have been shown to mediate analgesia induced by placebo,^57,58^ conditioned pain modulation,^59^ acupuncture,^60^ and hypnosis^61^ on several occasions. Significant reductions in pain-related brain activation (i.e., SI, posterior insula, parietal operculum) and activation in higher-order brain regions, such as the ACC, PFC, and insula, are associated with pain alleviation provided by these cognitive approaches.^62–67^ The PFC, insula, and ACC all have significant levels of opioid receptors and are linked to the production of analgesia via descending inhibitory networks.^68–71^ Meditation appears to decrease pain via involving brain regions with high concentrations of opioid receptors (sgACC, OFC, AI).^58,68,72^ In addition, mindfulness meditation lowers activation in the PAG, a brain area involved in opioid-mediated descending pain suppression.^73,74^ The findings of the present systematic review add to the growing body of data that mindfulness meditation reduces pain through mechanisms other than opioid dependent pathways, which is significant for chronic pain patients looking for a fast-acting nonopioid pain treatment.

Some of the identified studies in this review indicate that although the processes to promote mindfulness-based pain management may not directly correspond to reducing pain intensity, they do imply that mindfulness modifies the contextual appraisal of both pleasant and unpleasant sensory stimuli, and mindfulness meditation had a greater impact on the affective dimension of pain than on the sensory dimension, with the unpleasantness dimension of pain being considerably reduced with respect to pain intensity across some of the identified mindfulness/pain-focused experiments,^23–25,31^ which is a relevant impact when contemplating the use of meditation for clinical pain.

The research identified in this review, which were mostly conducted on healthy, pain-free people, have elucidated distinct pain-relieving mechanisms, such as cognitive and emotional reappraisal mechanisms, nonopioidergic pain modulatory pathways, acceptance in pain management, or enhanced activity in attentional control regions, which may now allow for the development of more effective mindfulness-based therapies to treat certain chronic pain problems. However, while the results from the majority of studies included in this systematic review have indeed provided valuable insights into the potential mechanisms through which mindfulness practices may influence pain perception, there are important limitations in generalizing these findings to patients with chronic pain conditions. First, it is crucial to acknowledge that pain experiences in healthy individuals and chronic pain patients are fundamentally different. In healthy subjects, pain typically serves as a protective mechanism, warning the body about potential or actual tissue damage. It is usually acute and short-lived, subsiding as the body heals. On the contrary, chronic pain persists or recurs over long periods, often beyond the normal healing period, and can be out of proportion to any observable injury or disease.^75^ It can also be associated with conditions where the nociceptive (pain sensing) system itself may be damaged, such as in neuropathic pain.^76^ Therefore, the neural mechanisms underlying acute pain in healthy individuals may differ significantly from those underlying chronic pain. Second, chronic pain is often associated with significant psychological distress and comorbid mental health conditions such as depression and anxiety.^77^ These can significantly modulate pain perception and response to pain management interventions, including mindfulness.^78,79^ The interplay of these psychological factors in the context of chronic pain is complex and may not be adequately reflected in studies involving healthy participants. Additionally, people living with chronic pain may have altered baseline brain structures and function due to the persistent pain experience. This includes changes in brain regions associated with pain modulation, such as the PFC and the ACC. These alterations could potentially affect the response to mindfulness interventions, as mindfulness practices are known to engage these areas and promote their FC.^10,48,49^ Pain experiences and coping strategies also can vary widely among individuals with chronic pain, depending on factors like the specific nature of their condition, its duration, their personal and social resources, and their previous experiences with pain management interventions.^80^ These variations add another layer of complexity to the generalizability of findings from healthy subjects to chronic pain patients.

Although the neuroscience of mindfulness-based pain management is still in its early stages, it has already demonstrated its ability to expand the known brain modulatory pain pathways and shed light on the complex affective and psychological aspects of pain management. The vast number of people suffering from chronic pain around the world highlight the potential value of developing validated self-administrable mind–body therapies that target pain in multiple ways via several distinct neuromodulatory pathways. However, due to the wide range of mindfulness meditation techniques and the biopsychosocial complexity of chronic pain conditions, future research should continue to use the highest experimental standards to investigate the clinically relevant treatment protocols to achieve long-term improvements in chronic pain management. Mindfulness-based techniques may play a role in changing the meaning, interpretation, and assessment of nociceptive information on its path to creating the subjective experience of pain.

Because the majority of the identified studies were conducted in healthy participants, more brain imaging and neurophysiological research is needed to delineate the processes through which mindfulness affects chronic pain. Hence, the results of these studies may not be transferable to the chronic pain population. In particular for chronic pain populations, there needs to be a better understanding of the brain pathways that promote mindfulness-based pain treatment. The brain processes that promote pain alleviation in chronic headache, for example, may differ from those that have been demonstrated to be beneficial in treating back pain.

Additionally, mindfulness meditation may be prone to nonspecific effects, i.e., slow breathing, demand characteristics, beliefs related to practicing meditation, conditioning, or posture, hence, placebo/sham-based comparisons are strongly recommended to better separate real mindfulness mechanisms.^16^ In the current review, only three studies^23,30,32^ using a placebo/sham control as a replacement for mindfulness meditation were identified, which highlights the scarcity of these approaches and the need for more rigorous future research in this area. Because three of the studies lacked a matched and active control group,^21,24,25^ it was difficult to determine if mindfulness lowers pain through brain pathways similar to placebo analgesia or other nonspecific influences.

Furthermore, the vast range of potential between-study and between-group variations (e.g., demographics, study characteristics, and meditation protocols) across the identified studies limits their generalizability to specific populations or treatment protocols.

## Conclusion

The research reviewed herein underscores mindfulness meditation as a multifaceted approach to pain management, revealing its efficacy through cognitive and emotional reappraisal mechanisms, nonopioidergic pain modulatory pathways, and enhanced activity in attentional control regions. Notably, mindfulness practices foster pain acceptance and alter perceptions of control over pain, indicating a comprehensive influence on both neurological and experiential aspects of pain. Despite these promising findings, the studies’ methodological limitations, including a focus on healthy individuals and varying experimental designs, call for cautious interpretation. This review highlights the need for future research to delve deeper into understanding mindfulness’s impact on chronic pain and exploring the mechanisms in populations with chronic pain conditions. Future studies should also differentiate the specific contributions of mindfulness techniques from nonspecific effects and extend investigations to clinical populations to confirm mindfulness-based interventions’ applicability and efficacy in managing chronic pain.

## Data Availability

The data of this systematic review can be provided by the corresponding author on reasonable request.
